# Association between admission pan-immune-inflammation value and short-term mortality in septic patients: a retrospective cohort study

**DOI:** 10.1038/s41598-024-66142-6

**Published:** 2024-07-02

**Authors:** Hong-Bo Xu, Yu-Hong Xu, Ying He, Xiao-Hua Lin, Zhijun Suo, Huaqing Shu, Haigang Zhang

**Affiliations:** 1 Department of Critical Care Medicine, Huazhong University of Science and Technology Union Shenzhen Hospital/The 6th Affiliated Hospital of Shenzhen University Health Science Center, 89 Taoyuan Road, Shenzhen, 518052 China; 2 Department of Pharmacy, The Eighth Affiliated Hospital, Sun Yat-Sen University, Shenzhen, 518033 China; 3 Department of Laboratory Medicine, The Eighth Affiliated Hospital, Sun Yat-Sen University, Shenzhen, 518033 China; 4 Department of Critical Care Medicine, Union Hospital, Tongji Medical College, Huazhong University of Science and Technology, 1277, Jiefang Avenue, Wuhan, 430022 China

**Keywords:** Pan-immune-inflammation value, Sepsis, Mortality, Inflammation, MIMIC-IV, Prognostic markers, Infectious diseases, Risk factors

## Abstract

Pan-Immune-Inflammation Value (PIV) has recently received more attention as a novel indicator of inflammation. We aimed to evaluate the association between PIV and prognosis in septic patients. Data were extracted from the Medical Information Mart for Intensive Care IV database. The primary and secondary outcomes were 28-day and 90-day mortality. The association between PIV and outcomes was assessed by Kaplan–Meier curves, Cox regression analysis, restricted cubic spline curves and subgroup analysis. A total of 11,331 septic patients were included. Kaplan–Meier curves showed that septic patients with higher PIV had lower 28-day survival rate. In multivariable Cox regression analysis, log2-PIV was positively associated with the risk of 28-day mortality [HR (95% CI) 1.06 (1.03, 1.09), *P* < 0.001]. The relationship between log2-PIV and 28-day mortality was non-linear with a predicted inflection point at 8. To the right of the inflection point, high log2-PIV was associated with an increased 28-day mortality risk [HR (95% CI) 1.13 (1.09, 1.18), *P* < 0.001]. However, to the left of this point, this association was non-significant [HR (95% CI) 1.01 (0.94, 1.08), *P* = 0.791]. Similar results were found for 90-day mortality. Our study showed a non-linear relationship between PIV and 28-day and 90-day mortality risk in septic patients.

Sepsis remains a major cause of mortality and critical illness worldwide, despite some advances in the sepsis pathophysiology and management of sepsis^1^. There were 48.9 million cases of sepsis worldwide in 2017, with 11 million sepsis-related deaths, accounting for 19.7% of all global deaths^2^. Sepsis has been identified as a global health priority by the World Health Organization (WHO)^3^. Early recognition of septic patients at high risk of poor prognosis is valuable for timely and appropriate management, resulting in improved patient outcomes^1,4^. Several scoring systems have been used to identify septic patients with poor outcomes. However, the use of them is inconvenient due to the inclusion of too many indicators, especially in clinical settings with limited medical conditions, such as the emergent department. Therefore, it remains important to find routinely available biomarkers for the early identification of septic patients with poor outcome.

The early dysregulated systemic inflammatory and immune response is considered a hallmark of sepsis. Markers associated with inflammation and immune status may have potential value in discriminating septic patients with poor prognosis^5^. Recently, a novel index, called Pan-Immune-Inflammation Value (PIV), has been reported as a comprehensive measure of host inflammatory and immune status^6^. This novel index was calculated from the following formula: neutrophil count × platelet count × monocyte count/lymphocyte count, all of which could be easily obtained from complete blood count test. In previous studies, the PIV index has shown promise in identifying patients with poor prognosis in a variety of inflammation- and immune-related diseases, such as myocardial infarction^7^, hypertension^8^, malignancy^9^, antineutrophil cytoplasmic antibody-associated vasculitis^10^, idiopathic pulmonary fibrosis^11^. Given this background, it seems reasonable to speculate about a possible association between PIV and sepsis mortality. However, to our knowledge, there are no reported studies on this topic. Therefore, in the present study, we aimed to investigate the association between PIV and short-term mortality in critical septic patients.

## Methods

### Sources of data

This study was based on a publicly available critical care database named the Multiparameter Intelligent Monitoring in Intensive Care (MIMIC) IV database (version 2.1). MIMIC IV contains deidentified ICU patient data from Beth Israel Deaconess Medical Center between 2008 and 2019^12^. The Institutional Review Boards of Beth Israel Deaconess Medical Center and the Massachusetts Institute of Technology approved this database. This study was reported following the Strengthening the Reporting of Observational Studies in Epidemiology guidelines^13^. Additionally, this study was exempt from the approval from our institutional review board (IRB) because it was based on a public deidentified database with the pre-existing IRB approval. Clinical data were extracted by HB Xu (35,959,043), who was granted access to the database after successfully completing an online training course.

### Participants

Adult septic patients were initially included according to the Sepsis 3.0 criteria. Patients were excluded they met any of the following criteria: (a) non-first ICU admission; (b) without data of neutrophil, platelet, monocyte, or lymphocyte counts within the first day of ICU admission; (c) age < 18 years; (d) length of ICU stay or hospital stay less than 24 h; (e) diagnosed with malignancy (including haematological neoplasms), human immunodeficiency virus (HIV) infection or acquired immunodeficiency syndrome(AIDS).

### Data extraction

Data on demographic parameters, comorbidities, vital signs, laboratory parameters, scoring systems and supportive therapies were extracted from MIMIC IV using PostgreSQL software (version 9.6). Demographic parameters included age, ethnicity and sex. Comorbidities included hypertension, diabetes mellitus, myocardial infarct, congestive heart failure, chronic pulmonary disease, severe liver disease, chronic renal disease and cerebrovascular disease. Vital signs included temperature, respiratory rate, heart rate, mean arterial pressure (MAP) and pulse oxygen saturation (SpO2). Laboratory parameters included white blood cell (WBC), hemoglobin, red blood cell distribution width (RDW), glucose, serum creatinine, blood urea nitrogen (BUN), anion gap, bicarbonate, sodium, potassium, prothrombin time, partial thromboplastin time, international normalized ratio (INR), lactate, neutrophils, platelets, monocytes and lymphocytes. Scoring systems included the Sequential Organ Failure Assessment (SOFA) Score and the Simplified Acute Physiology Score II (SAPS II). Supportive therapies included the use of renal replacement therapy (RRT), mechanical ventilation, and vasoactive agents during the first day after ICU admission. Comorbidities were diagnosed according to International Classification of Diseases (ICD)-9 and ICD-10 codes. It was difficult to identify patients with septic shock in this database due to a lack of relevant information. Thus, data on the use of vasoactive agents were extracted for subgroup analysis. If a laboratory parameter was tested multiple times during the 24-h of ICU stay, the first value was extracted for analysis. The PIV index was calculated using the formula “neutrophil count (k/ul) × monocyte count (k/ul) × platelet count (k/ul)/lymphocyte count (k/ul)”^6^.

### Outcomes

The primary endpoint was 28-day all-cause mortality after ICU admission, and the secondary endpoint was 90-day all-cause mortality.

### Management of missing data and outliers

Missing data are common in the MIMIC IV database. The missing proportion of the extracted variables was less than 5%, except for lactate with a high missing proportion (24.6%). Details of missing values are shown in supplementary materials (Table S1). As that serum lactate is an important variable in sepsis, it was treated as a dummy variable, with a category to indicate the “missing” status. For the remaining variables with missing values, they were replaced by the respective mean or median value, where appropriate. Furthermore, variables with abnormal values were adjusted using the winsor2 command with replace cuts (1.99). The STATA software (version 14) was used to manage the missing data and outliers.

### Statistical analysis

PIV was log2 transformed before analysis due to its skewed distribution. Continuous variables were expressed as mean ± standard deviation (SD) or median (interquartile range) and compared using one-way analysis of variance or Kruskal–Wallis test. Categorical data were presented as numbers (percentages) and analyzed by chi-square or Fisher exact tests. Survival curves were estimated by Kaplan–Meier survival analysis and compared using log-rank test. The potential association between log2-PIV and the risk of 28-day and 90-day all-cause mortality were evaluated by unadjusted and multivariable-adjusted Cox proportional hazards models. In the multivariable analysis, adjusted covariates were selected on the basis of clinical relevance and a change in effect estimate greater than 10%^14,15^. The assumption of multicollinearity was also assessed, and a variance inflation factor (VIF) value greater than 5 indicated the presence of multicollinearity between independent variables. Model 1, non-adjusted; Model 2, adjusted for age, ethnicity and sex; Model 3, adjusted for age, ethnicity, sex, SOFA, SAPS II, mechanical ventilation, renal replacement therapy, vasoactive agents, myocardial infarct, congestive heart failure, hypertension, diabetes mellitus, chronic pulmonary disease, liver disease, chronic renal disease, cerebrovascular disease, temperature, respiratory rate, MAP, SPO2, WBC, hemoglobin, BUN, anion gap, bicarbonate, lactate, RDW, glucose, creatinine, INR. The shape between log2-PIV and survival endpoints was explored by restricted cubic spline analysis. If a non-linear association was found, a two-piecewise regression model was conducted to elucidate the non-linearity. Additionally, we examined the association between PIV and the risk of 28-day and 90-day mortality in several subgroups, including age (< 65 and ≥ 65 years), sex, race, myocardial infarct, congestive heart failure, hypertension, diabetes mellitus, chronic pulmonary disease, severe liver disease, chronic renal disease, cerebrovascular disease, RRT use, ventilator use, vasoactive drug use, SOFA (< 6 and ≥ 6), and SAPS II (< 40 and ≥ 40). Finally, we conducted two sensitivity analyses to verify the findings. Firstly, patients with missing values were excluded. Secondly, patients with autoimmune diseases, including systemic lupus erythematosus, systemic sclerosis, ulcerative colitis, rheumatoid arthritis, ankylosing spondylitis, Crohn's disease, multiple sclerosis, myasthenia gravis and polymyositis were removed, because of their potential effect on WBC counts^16^. Data analyses were performed using R 4.2.1 (http://www.R-project.org) and Free Statistics software (version 1.7.1, Free Clinical Medical Technology Co., Ltd, Beijing, China). A two-tailed *P* value < 0.05 was considered statistically significant.

### Institutional review board statement

The use of MIMIC database was approved by the Institutional Review Board of the Massachusetts Institute of Technology and Beth Israel Deaconess Medical Center.

## Results

### Baseline characteristics

**Figure 1:**
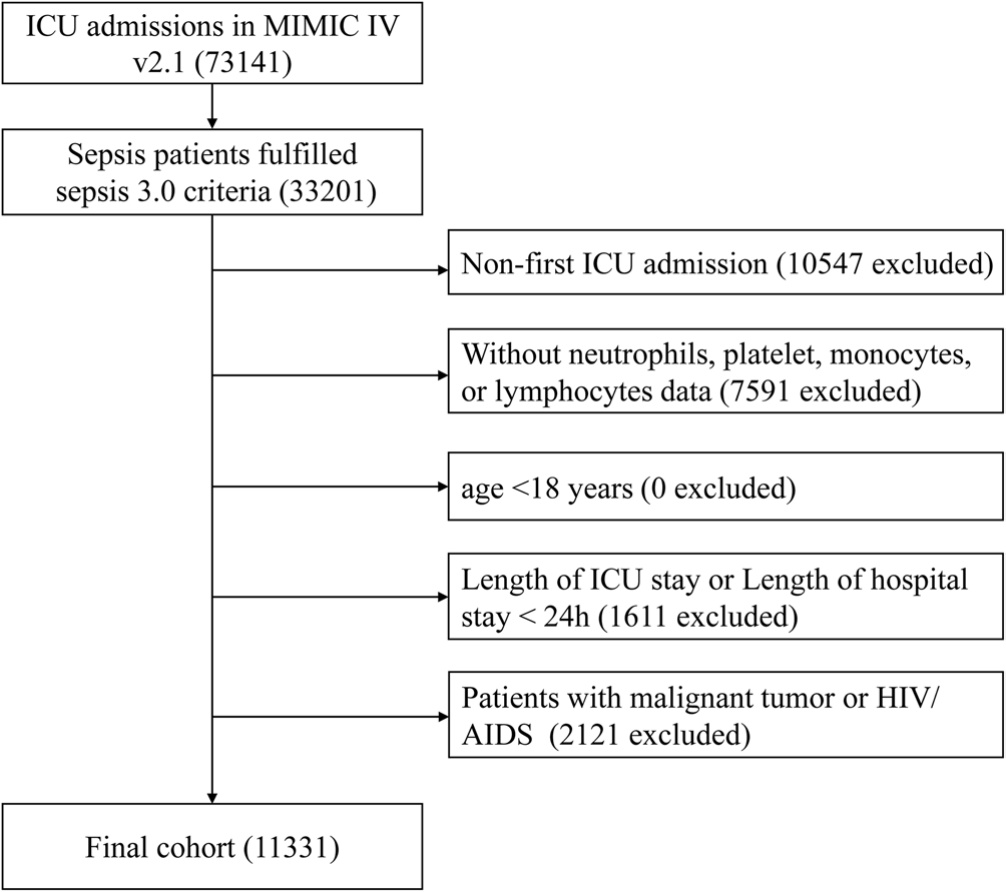
Flow chart of subject selection. ICU, intensive care unit; MIMIC, Medical Information Mart for Intensive Care; HIV, human immunodeficiency virus; AIDS, acquired immunodeficiency syndrome.

Figure 1 shows the patient screening process, and 11,331 patients were finally enrolled. The enrolled patients were divided into four groups according to the quartiles of log2-PIV (Q1: < 8.10, Q2: 8.10–9.63, Q3: 9.63–11.06, Q4: ≥ 11.06). Baseline characteristics are shown in Table 1. In general, the final cohort consisted of 6461 men and 4870 women with a mean age of 66.0 ± 16.9 years. Of the enrolled patients, 65.4% were white. Patients with higher log2-PIV had higher prevalence of comorbidities, including myocardial infarct, congestive heart failure, diabetes mellitus, chronic pulmonary disease and chronic kidney disease. Conversely, patients with lower log2-PIV were more likely to have a history of liver disease. Patients with higher log2-PIV had higher SAPS II scores. With increasing log2-PIV, there was an increasing trend in WBC, RDW, glucose, creatinine, blood urea nitrogen and anion gap. In the study population, the 28-day and 90-day mortality were 17.2% and 22.5%, respectively. Compared to patients with lower log2-PIV, those with higher log2-PIV had higher 28-day and 90-day mortality.

**Table 1:** Basic characteristics of included patients grouped according to Log2-PIV quartiles.

Variables	Q1 (< 8.10) (n = 2817)	Q2 (≥ 8.10, < 9.63) (n = 2832)	Q3 (≥ 9.63, < 11.06) (n = 2841)	Q4 (≥ 11.06) (n = 2841)	*P*-value
Age (years)	64.8 ± 16.1	66.2 ± 16.5	66.0 ± 17.6	67.0 ± 17.4	< 0.001
Sex, male, n (%)	1647 (58.5)	1594 (56.3)	1592 (56.0)	1628 (57.3)	0.236
Race, white, n (%)	1827 (64.9)	1842 (65)	1859 (65.4)	1888 (66.5)	0.59
Comorbidities, n (%)					
Myocardial infarct	436 (15.5)	490 (17.3)	568 (20)	610 (21.5)	< 0.001
Congestive heart failure	719 (25.5)	817 (28.8)	976 (34.4)	1039 (36.6)	< 0.001
Hypertension	1771 (62.9)	1849 (65.3)	1835 (64.6)	1856 (65.3)	0.179
Diabetes mellitus	841 (29.9)	867 (30.6)	937 (33)	913 (32.1)	0.047
Chronic pulmonary disease	657 (23.3)	711 (25.1)	802 (28.2)	837 (29.5)	< 0.001
Liver disease	574 (20.4)	481 (17)	397 (14)	364 (12.8)	< 0.001
Chronic renal disease	524 (18.6)	609 (21.5)	637 (22.4)	719 (25.3)	< 0.001
Cerebrovascular disease	337 (12)	474 (16.7)	536 (18.9)	401 (14.1)	< 0.001
Vital signs					
Temperature, °C	36.6 (36.1, 37.0)	36.7 (36.3, 37.1)	36.8 (36.4, 37.2)	36.8 (36.4, 37.2)	< 0.001
Heart rate, beats/min	83.0 (74.0, 97.0)	85.0 (75.0, 99.0)	90.0 (77.0, 104.0)	94.0 (80.0, 109.0)	< 0.001
Respiratory rate, beats/min	17.0 (14.0, 21.0)	18.0 (15.0, 22.0)	19.0 (16.0, 24.0)	20.0 (17.0, 25.0)	< 0.001
MAP, mmHg	79.0 (69.0, 90.0)	81.0 (70.0, 93.0)	82.0 (70.0, 94.0)	81.0 (70.0, 94.0)	< 0.001
SPO2	100.0 (96.0, 100.0)	99.0 (96.0, 100.0)	98.0 (95.0, 100.0)	97.0 (94.0, 100.0)	< 0.001
Laboratory tests					
WBC (k/ul)	7.5 (5.3, 10.3)	10.4 (8.1, 13.6)	12.9 (10.3, 16.3)	18.2 (14.3, 23.5)	< 0.001
HGB (g/dL)	10.4 ± 2.4	11.2 ± 2.5	11.5 ± 2.4	11.5 ± 2.5	< 0.001
RDW (%)	14.2 (13.2, 15.6)	14.2 (13.3, 15.8)	14.3 (13.4, 15.7)	14.6 (13.5, 16.2)	< 0.001
Glucose (mg/dL)	121.0 (102.0, 151.0)	128.0 (105.0, 163.0)	134.0 (109.0, 179.0)	144.0 (114.0, 194.0)	< 0.001
Creatinine (mg/dL)	0.9 (0.7, 1.4)	1.1 (0.8, 1.6)	1.1 (0.8, 1.8)	1.2 (0.9, 2.0)	< 0.001
BUN (mg/dL)	19.0 (13.0, 29.0)	21.0 (14.0, 34.0)	23.0 (15.0, 39.0)	26.0 (17.0, 43.0)	< 0.001
Anion gap (mEq/L)	14.5 ± 5.0	15.5 ± 4.9	16.5 ± 4.9	17.9 ± 5.4	< 0.001
Bicarbonate (mEq/L)	22.5 ± 4.5	22.7 ± 4.9	22.4 ± 5.1	21.5 ± 5.7	< 0.001
Sodium (mEq/L)	138.9 ± 4.9	138.5 ± 5.7	138.0 ± 5.8	137.4 ± 6.3	< 0.001
Potassium (mEq/L)	4.2 ± 0.8	4.3 ± 0.8	4.4 ± 1.0	4.5 ± 1.0	< 0.001
INR	1.4 (1.2, 1.6)	1.3 (1.1, 1.6)	1.3 (1.1, 1.5)	1.3 (1.1, 1.6)	< 0.001
PT (second)	15.0 (13.1, 17.4)	14.2 (12.4, 17.0)	13.9 (12.3, 16.4)	14.3 (12.6, 17.1)	< 0.001
PTT (second)	31.6 (27.9, 37.7)	30.4 (26.7, 35.9)	29.9 (26.4, 35.6)	30.2 (26.5, 35.8)	< 0.001
Lactate (mmol/L)					< 0.001
≤ 4	1744 (61.9)	1620 (57.2)	1643 (57.8)	1698 (59.8)	
> 4	492 (17.5)	427 (15.1)	416 (14.6)	504 (17.7)	
Missing	581 (20.6)	785 (27.7)	782 (27.5)	639 (22.5)	
Neutrophils (k/ul)	5.3 (3.4, 7.7)	8.3 (6.3, 11.1)	10.9 (8.6, 13.8)	16.0 (12.6, 20.4)	< 0.001
Platelet (k/ul)	129.0 (92.0, 179.0)	175.0 (130.0, 230.0)	215.0 (163.0, 279.0)	261.0 (196.0, 351.0)	< 0.001
Monocytes (k/ul)	0.2 (0.1, 0.4)	0.5 (0.3, 0.6)	0.6 (0.4, 0.8)	1.0 (0.7, 1.3)	< 0.001
Lymphocytes (k/ul)	1.4 (0.8, 2.1)	1.3 (0.8, 2.0)	1.1 (0.7, 1.6)	0.8 (0.5, 1.3)	< 0.001
PIV	122.5 (61.6, 195.8)	485.3 (369.1, 629.1)	1274.0 (1003.0, 1631.0)	4166.0 (2878.0, 7073.0)	< 0.001
Log2-PIV	6.5 ± 1.5	8.9 ± 0.4	10.3 ± 0.4	12.3 ± 1.0	< 0.001
Disease severity scores					
SOFA	6.0 (4.0, 9.0)	6.0 (4.0, 9.0)	6.0 (4.0, 9.0)	6.0 (4.0, 9.0)	< 0.001
SAPS II	36.0 (28.0, 45.0)	37.0 (29.0, 46.0)	38.0 (30.0, 47.0)	40.0 (32.0, 50.0)	< 0.001
Support therapies (n, %)					
Mechanical ventilation	1562 (55.4)	1531 (54.1)	1496 (52.7)	1503 (52.9)	0.135
Renal replacement treatment	76 (2.7)	83 (2.9)	97 (3.4)	142 (5)	< 0.001
Vasoactive agents	1557 (55.3)	1332 (47)	1201 (42.3)	1330 (46.8)	< 0.001
Outcomes (n, %)					
28-day mortality	368 (13.1)	389 (13.7)	509 (17.9)	686 (24.1)	< 0.001
90-day mortality	473 (16.8)	529 (18.7)	675 (23.8)	872 (30.7)	< 0.001

Continuous variables are presented as the mean (standard deviation) or median (interquartile range), while categorical variables are presented as numbers (proportions). PIV, pan-immune-inflammation value; MAP, mean blood pressure; SPO2, peripheral capillary oxygen saturation; WBC, white blood cell; HGB, hemoglobin; RDW, red cell distribution width; BUN, blood urea nitrogen; INR, international normalized ratio; PT, prothrombin time; PTT, partial thromboplastin time; SOFA, Sequential Organ Failure Assessment; SAPS II, Simplified Acute Physiology Score II.

### Association between PIV and short-term mortality

**Figure 2:**
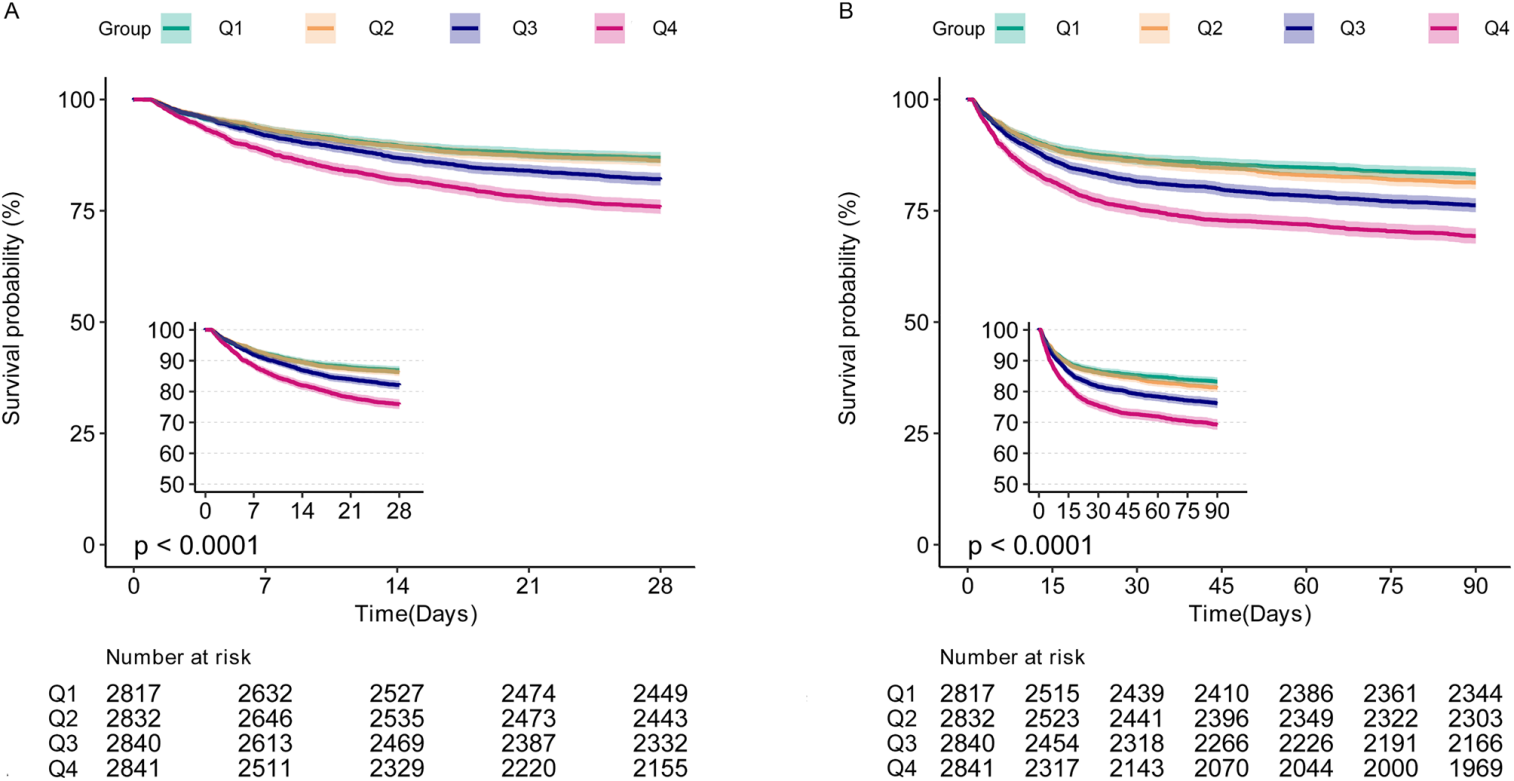
The 28-day (**A**) and 90-day (**B**) survival curves in septic patients stratified by quartiles of log2-PIV. PIV, Pan-Immune-Inflammation Value. Log2-PIV index: Q1: < 8.10, Q2: 8.10–9.63, Q3: 9.63–11.06, Q4: ≥ 11.06.

The cumulative 28-day all-cause mortality rate was assessed according to log2-PIV quartiles, and it was shown that septic patients with higher log2-PIV had a significantly lower 28-day survival rate (*P* for log-rank test < 0.0001) (Fig. 2A). Similar results were found for cumulative 90-day all-cause mortality rate (*P* for log-rank test < 0.0001) (Fig. 2B).

In addition, several Cox regression models were performed to assess the association between log2-PIV and prognostic outcomes in septic patients (Table 2). It was found that a higher log2-PIV was associated with an increased risk of 28-day all-cause mortality (model 1: hazard ratio [HR] = 1.12, 95% confidence interval [CI] 1.10–1.41, *P* < 0.001; model 2: HR = 1.11, 95% CI 1.09–1.14, *P* < 0.001; model 3: HR = 1.06, 95% CI 1.03–1.09, *P* < 0.001). We also analyzed log2-PIV as a categorical variable based on quartiles. Compared with the Q1 group, a higher log2-PIV group (Q4) was associated with an increased risk of 28-day mortality (model 1: HR = 1.96, 95% CI 1.73–2.22, *P* < 0.001; model 2: HR = 1.88, 95% CI 1.65–2.13, *P* < 0.001; model 3: HR = 1.49, 95% CI 1.26–1.75, *P* < 0.001). A similar trend was observed in Q3 group. However, the 28-day mortality risk did not differ between the Q2 and Q1 groups (model 1: HR = 1.05, 95% CI 0.91–1.21, *P* = 0.484; model 2: HR = 1.01, 95% CI 0.88–1.17, *P* = 0.873; model 3: HR = 0.98, 95% CI 0.84–1.13, *P* = 0.758). Similar results were found when investigating the association between log2-PIV and 90-day all-cause mortality (Table 2).

**Table 2:** Multivariable results by Cox regression analysis.

	Model 1		Model 2		Model 3	
	HR (95% CI)	*P* value	HR (95% CI)	*P* value	HR (95% CI)	*P* value
28-day mortality						
Log2-PIV	1.12 (1.10–1.14)	< 0.001	1.11 (1.09–1.14)	< 0.001	1.06 (1.03–1.09)	< 0.001
Quartile						
Q1	Reference		Reference		Reference	
Q2	1.05 (0.91–1.21)	0.484	1.01 (0.88–1.17)	0.873	0.98 (0.84–1.13)	0.758
Q3	1.40 (1.22–1.60)	< 0.001	1.35 (1.18–1.55)	< 0.001	1.20 (1.04–1.39)	0.014
Q4	1.96 (1.73–2.22)	< 0.001	1.88 (1.65–2.13)	< 0.001	1.49 (1.26–1.75)	< 0.001
* P* for trend		< 0.001		< 0.001		< 0.001
90-day mortality						
Log2-PIV	1.12 (1.10–1.14)	< 0.001	1.11 (1.09–1.13)	< 0.001	1.08 (1.05–1.11)	< 0.001
Quartile						
Q1	Reference		Reference		Reference	
Q2	1.12 (0.99–1.26)	0.08	1.07 (0.95–1.21)	0.282	1.03 (0.91–1.18)	0.609
Q3	1.46 (1.30–1.64)	< 0.001	1.41 (1.26–1.59)	< 0.001	1.28 (1.13–1.46)	< 0.001
Q4	1.98 (1.77–2.22)	< 0.001	1.90 (1.70–2.13)	< 0.001	1.58 (1.37–1.83)	< 0.001
* P* for trend		< 0.001		< 0.001		< 0.001

Model 1 adjusted for none; Model 2 adjusted for age, sex, and race; Model 3 adjusted for age, sex, race, SOFA, SAPS II, mechanical ventilation, renal replacement treatment, vasoactive agents, myocardial infarct, congestive heart failure, hypertension, diabetes mellitus, chronic pulmonary disease, liver disease, chronic renal disease, cerebrovascular disease, temperature, respiratory rate, MAP, SPO2, WBC, HGB, BUN, anion gap, bicarbonate, Lactate, RDW, glucose, creatinine, INR. PIV, pan-immune-inflammation value; SOFA, Sequential Organ Failure Assessment; SAPS II, Simplified Acute Physiology Score II; MAP, mean blood pressure; SPO2, peripheral capillary oxygen saturation; WBC, white blood cell; HGB, hemoglobin; RDW, red cell distribution width; BUN, blood urea nitrogen; INR, international normalized ratio.

**Figure 3:**
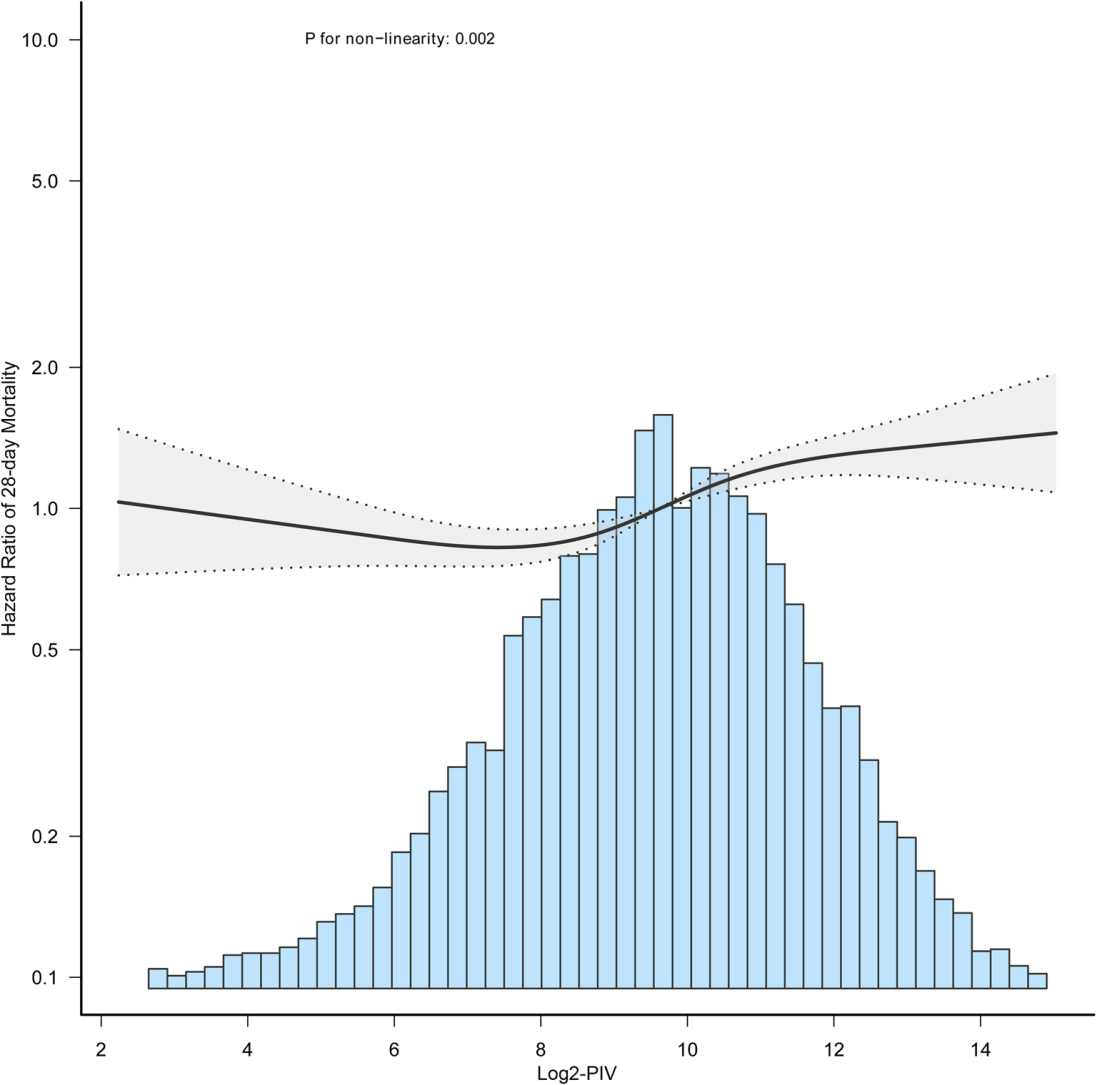
Association between Log2-PIV and 28-day mortality in septic patients. Data were fitted by a multivariable-adjusted restricted cubic spline Cox’s regression. A non-linear association between Log2-PIV and 28-day mortality was observed. Log2-PIV was entered as a continuous variable, the variables in model 3 of Table 2 were adjusted. The curves line and shaded ribbons around represented the estimated values and their corresponding 95% confidence intervals. PIV, Pan-immune-inflammation value.

Furthermore, the shape of the relationship between log2-PIV and mortality risk was assessed using restricted cubic spline regression. After adjustment for confounders, there was a non-linear relationship between log2-PIV and 28-day mortality in septic patients (Fig. 3). Further analysis by a two-piecewise linear regression model revealed that the inflection point of log2-PIV was 8 (Table 3). To the left of this point (log2-PIV < 8), there was no significant association between log2-PIV and 28-day mortality [HR (95% CI) 1.01 (0.94, 1.08), P = 0.791]. To the right of the inflection point (log2-PIV ≥ 8), we found that log2-PIV was positively associated with 28-day mortality [HR (95% CI) 1.13 (1.09, 1.18), P < 0.001]. A similar non-linear relationship between log2-PIV and 90-day mortality was also found (Figure S1, table S2).

**Table 3:** Threshold effect analysis of the association between Log2-PIV and 28-day mortality.

	HR (95% CI)	*P* value
Standard Cox regression model	1.06 (1.03–1.09)	< 0.001
Two-piecewise Cox regression model		
< 8	1.01 (0.94, 1.08)	0.791
≥ 8	1.13 (1.09, 1.18)	< 0.001
* P* for the log likelihood ratio test		0.014

Variables included in model 3 (Table 2) were adjusted.

### Subgroup analysis

Results of subgroup analyses for the association between log2-PIV and 28-day mortality are presented in supplementary materials (Figure S2). There were no significant interactions across subgroups of sex, race, myocardial infarct, congestive heart failure, diabetes mellitus, chronic pulmonary disease, chronic renal disease, cerebrovascular disease, use of RRT, use of mechanical ventilation, and use of vasoactive agents (all* P* for interaction > 0.05). There were significant interactions in other subgroups, including age, hypertension, severe liver disease, SOFA and SASP II (all* P* for interaction < 0.05). Older septic patients (≥ 65 years) had higher 28-day mortality with increasing log2-PIV. The HRs (95% CI) for Q2, Q3, and Q4 were 1.08 (0.89–1.31), 1.36 (1.13–1.64), and 1.81 (1.48–2.22), respectively, with Q1 as the reference group. Septic patients with hypertension had a higher risk of 28-day mortality [Q2: HR (95% CI) 0.93 (0.77–1.13); Q3: HR (95% CI) 1.3 (1.07–1.56); Q4: HR (95% CI) 1.82 (1.48–2.24)]. Patients without severe liver disease showed a higher 28-day mortality risk [Q2: HR (95% CI) 0.94 (0.78–1.12); Q3: HR (95% CI) 1.15 (0.97–1.37); Q4: HR (95% CI) 1.58 (1.31–1.91)]. Additionally, we found septic patients with lower SOFA (< 6) [Q2: HR (95% CI) 1.18 (0.8–1.74); Q3: HR (95% CI) 1.77 (1.21–2.59); Q4: HR (95% CI) 2.58 (1.7–3.94)] and lower SAPS II score (< 40) [Q2: HR (95% CI) 1.05 (0.79–1.39); Q3: HR (95% CI) 1.53 (1.16–2.02); Q4: HR (95% CI) 1.67 (1.19–2.33)] were associated with a higher 28-day mortality risk. In the subgroup analyses of 90-day mortality, similar results were found (Figure S3).

### Sensitivity analysis

The results of the two sensitivity analyses are presented in the supplementary materials. After excluding patients with missing values, the association between log2-PIV and 28-day and 90-day mortality remained stable (Table S3). Furthermore, when patients with autoimmune diseases were excluded, the associations remained strong (Table S4).

## Discussion

There were two main findings from this study. Firstly, higher log2-PIV was significantly associated with increased risk of 28-day and 90-day mortality in septic patients. Secondly, log2-PIV showed a non-linear relationship with 28-day and 90-day mortality risk with an inflection point of 8. To the right of this point, log2-PIV was positively related to 28-day/90-day mortality. However, to the left of this point, there was no significant association between them.

Systemic immune dysregulation is involved in the pathophysiology of sepsis, which can be partially indicated by peripheral blood cells, such as neutrophils, lymphocytes, platelets and monocytes^17,18^. Neutrophils, as key players in the innate immune system, act a crucial role in the containment and eradication of microbes and in protecting the host from infection. A significant increase in neutrophil count has been observed in severe infectious diseases, particularly in cases of sepsis, as an indicator of the severity of infection. During sepsis, neutrophils undergo functional changes, including delayed apoptosis, increased immature neutrophils, altered antimicrobial activity and impaired neutrophil recruitment and migration^19^. Collectively, these changes reduce the rate of pathogen clearance, increase susceptibility to secondary infection and ultimately lead to a poor prognosis. Monocytes are involved in both innate and adaptive immune responses to pathogens through multiple mechanisms, including phagocytosis, neutrophil recruitment, release of reactive oxygen species and inflammatory cytokines, antigen presentation and activation of lymphocytes^20^. This functional diversity is supported by the heterogeneity of the monocyte subpopulation. During sepsis, monocytes are activated by pattern recognition receptors (PRRs) and sepsis-associated hypoxia, leading to changes in monocyte count, subpopulation ratios and functional characteristics^21,22^. In addition to their well-known role in hemostasis and coagulation, platelets have been recognized as important players in the inflammatory response of sepsis, by inducing the release of cytokines, releasing microbicidal molecules and interacting with immune cells, including neutrophils, lymphocytes, and monocytes^23^. Lymphocytes are the key cell type in the adaptive immune response. Sepsis-induced lymphocyte apoptosis leads to lymphocyte depletion, which is an important cause of sepsis-related immunosuppression^24^. Generally, each cell type not only performs its own function but also works together to reflect the immune status of host. Additionally, the counts of different blood cells can be altered in several physical and pathological conditions, including age, gender, obesity, hypertension, smoking, hemopathies, and inflammatory non-infectious diseases. Therefore, the interpretation of the impact of differential blood cell counts on sepsis cannot be solely based on them^17^.

More recently, PIV, an indicator that integrates neutrophil, platelet, monocyte and lymphocyte count, was first reported as a novel systematic inflammatory indicator by Giovanni Fuca et al. in 2020^6^. PIV was shown to be a significant predictor of survival outcomes, outperforming other inflammatory biomarkers in patients with metastatic colorectal cancer. Due to its potential to comprehensively represent a patient’s immunity and systemic inflammation, PIV has recently received more attention from researchers. Subsequently, PIV has been shown to be a promising prognostic marker in various cancers, as reviewed by Guven et al.^9^. Murat and colleagues reported that PIV was a better indicator for predicting one-month and one-year all-cause mortality in patients with ST-segment elevation myocardial infarction (STEMI)^7^. Hypertensive patients with a high PIV had an increased risk of all-cause mortality and cardiovascular mortality^8^. The key role of immune dysfunction in the pathophysiology of sepsis led us to associate PIV with the prognosis of septic patients. In the present study, Cox regression analysis displayed that high PIV was an independent risk factor for 28-day and 90-day all-cause mortality in critical septic patients. Further sensitivity analyses showed that the results remained consistent, indicating the reliability of our findings. Additionally, we found that log2-PIV had a non-linear relationship with the clinical outcomes, with a predicted inflection point at 8.0. To the right of the threshold, the 28-day mortality increased as log2-PIV increased. To the left of the threshold, there was no significant association between log2-PIV and 28-day mortality.

It is important to note that because the PIV is calculated using platelet counts in the numerator, a lower platelet count results in a lower PIV. Thrombocytopenia is common in sepsis and has been recognized as an independent risk factor for mortality in sepsis. However, some researchers did not find such association or only found an inconstant effect depending on the clinical status of the patient^25^. Additionally, it was reported that the platelet counts often decreased in the first days after ICU admission and reached a nadir around day 4 in critical ill patients^26^. Therefore, investigation of the dynamic changes in PIV may be more valuable in predicting sepsis prognosis. Further studies are needed.

According to the stratified analysis, PIV had a stronger prognostic value in septic patients with lower SOFA score (< 6) and SAPS II score (< 40). However, the trend of the effect size was consistent in these subgroups. This finding suggests that septic patients with mild organ dysfunction (SOFA < 6) may have a higher risk of mortality if they have a higher PIV, indicating the existence of a subgroup of sepsis patients who require closer attention despite their lower SOFA scores. Interestingly, similar results have been reported in other studies evaluating the association between inflammatory markers and sepsis mortality. It was reported that the association between a high neutrophil-to-lymphocyte ratio and the risk of 28-day mortality was more pronounced in septic patients with lower SOFA score (< 5) and SAPS II score (< 40)^27^. Shen and coworkers showed that the significant association between high platelet-to-lymphocyte ratio and in-hospital mortality risk was only found in septic patients with a SOFA score < 10^28^. Several factors may explain the observed interaction effects. Critically ill septic patients who have higher SOFA score may already have a higher baseline mortality risk, and more severe organ dysfunction may complicate sepsis management, leading to less optimal treatment and worse outcomes. These may attenuate the association between PIV and mortality in septic patients with higher SOFA scores. Age (≥ 65 years) also showed an interaction with PIV in 28-day mortality, suggesting that PIV may be an important biomarker in predicting prognosis in elderly septic patients. The aging process is associated with changes in the immune system, leading to significant differences in the immune system between elderly individuals and young or middle-aged adults^29^. Aging is accompanied by chronic, low-grade inflammation which makes the elderly population more susceptible to infection and at higher risk of mortality. In addition, our results suggested that the prognostic value of PIV is more significant in septic patients with hypertension. Patients with hypertension are accompanied with inflammation activation^30,31^. Recently, PIV was reported to be positively associated with long-term all-cause mortality and cardiovascular mortality in hypertensive patients^8^. Furthermore, we found that PIV had a weaker prognostic value in septic patients with severe liver disease. Similar to our results, Jiang and colleagues also reported that the prognostic value of the systemic immune-inflammation index appeared to be attenuated in patients with sepsis and severe liver disease^32^. Liver disease is associated with a wide range of haematological abnormalities, including platelet thrombocytopenia^33^. Additionally, the balance between immune tolerance and effective immune responses has been suggested to be impaired in severe liver disease, and this immune dysregulation may affect the coordinated response of the liver to infection^34^. Finally, severe liver disease itself can increase the mortality in septic patients, with mortality rates as high as 54–68%^35^. These may influence the relationship between PIV and prognosis in septic patients with liver disease. In general, our study is the first to report these interactions. The specific mechanisms are still unclear. Further studies are requited.

Some limitations should be acknowledged. Firstly, due to the limitations of a retrospective study, residual confounding factors are difficult to avoid although we did our best to adjust for confounders. Secondly, PIV was only evaluated at ICU admission, as our main aim was to evaluate the potential of PIV in the early identification of septic patients with poor prognosis. Future studies focusing on the dynamic changes of PIV during hospital stay are needed. Thirdly, the potential selection bias in a single center study should be acknowledged, which may limit the generalisability of our results. Therefore, further prospective multicenter studies are required to verify our results in the future.

## Conclusions

PIV showed a non-linear relationship with 28-day and 90-day mortality risk in septic patients. PIV may be a simple, inexpensive and convenient marker for the early identification of septic patients with poor prognosis.

### Supplementary Information

Supplementary Information.

## Data Availability

The data used to support the findings of this study can be obtained from the MIMIC-IV database (https://mimic.physionet.org/).
