# Supplementary material for: Association between admission pan-immune-inflammation value and short-term mortality in septic patients: a retrospective cohort study
Source: Sci Rep. 2024 Jul 2;14:15205. doi: 10.1038/s41598-024-66142-6 (PMC11219806; doi:10.1038/s41598-024-66142-6)

**Supplementary materials**

Table S1. The proportion of missing values for the extracted variables

Table S2. Threshold effect analysis of the association between Log2-PIV and 90-day mortality.

Table S3. Multivariable results by Cox regression analysis (sensitivity analysis 1)

Table S4. Multivariable results by Cox regression analysis (sensitivity analysis 2)

Figure S1. Association between Log2-PIV and 90-day mortality in septic patients. Data were ﬁtted by a multivariable-adjusted restricted cubic spline Cox’s regression. A non-linear association between Log2-PIV and 90-day mortality was observed. Log2-PIV was entered as a continuous variable, the variables in model 3 of table 2 were adjusted. The curves line and shaded ribbons around represented the estimated values and their corresponding 95% conﬁdence intervals. PIV, Pan-Immune-Inflammation Value.

Figure S2. Forest plot for subgroup analysis of the association between Log2-PIV and 28-day mortality in sepsis patients. PIV, Pan-Immune-Inflammation Value; SAPS II, Simpliﬁed Acute Physiology Score II; SOFA, Sequential Organ Failure Assessment; HR, hazard ratio; CI, confidence interval.

Figure S3. Forest plot for subgroup analysis of the association between Log2-PIV and 90-day mortality in sepsis patients. PIV, Pan-Immune-Inflammation Value; SAPS II, Simpliﬁed Acute Physiology Score II; SOFA, Sequential Organ Failure Assessment; HR, hazard ratio; CI, confidence interval.

Table S1. The proportion of missing values for the extracted variables

| Variables | Missing data (n) | Missing data (%) |
| --- | --- | --- |
| Age | 0 | 0 |
| Sex | 0 | 0 |
| Race | 0 | 0 |
| Myocardial infarct | 0 | 0 |
| Congestive heart failure | 0 | 0 |
| Hypertension | 0 | 0 |
| Diabetes mellitus | 0 | 0 |
| Chronic pulmonary disease | 0 | 0 |
| Liver disease | 0 | 0 |
| Chronic renal disease | 0 | 0 |
| Cerebrovascular disease | 0 | 0 |
| Temperature ,℃ | 93 | <1% |
| Heart rate, beats/min | 5 | <1% |
| Respiratory rate, beats/min | 45 | <1% |
| MAP, mmHg | 25 | <1% |
| SPO2 | 3 | <1% |
| White blood cell (k/ul) | 0 | 0 |
| Hemoglobin (g/dL) | 5 | <1% |
| Red cell distribution width (%) | 6 | <1% |
| Glucose (mg/dL) | 38 | <1% |
| Creatinine (mg/dL) | 1 | <1% |
| Blood urea nitrogen (mg/dL) | 2 | <1% |
| Anion gap (mEq/L) | 9 | <1% |
| Bicarbonate (mEq/L) | 1 | <1% |
| Sodium (mEq/L) | 2 | <1% |
| Potassium (mEq/L) | 5 | <1% |
| INR | 354 | 3.12% |
| PT (second) | 354 | 3.12% |
| PTT (second) | 395 | 3.48% |
| Lactate (mmol/L) | 2787 | 24.60% |
| Neutrophils (k/ul) | 0 | 0 |
| Platelet (k/ul) | 0 | 0 |
| Monocytes (k/ul) | 0 | 0 |
| Lymphocytes (k/ul) | 0 | 0 |
| SOFA | 0 | 0 |
| SAPS II | 0 | 0 |
| Mechanical ventilation | 0 | 0 |
| Renal replacement treatment | 0 | 0 |
| Vasoactive agents | 0 | 0 |

Table S2. Threshold effect analysis of the association between Log2-PIV and 90-day mortality.

|  | HR (95% CI) | *P* value |
| --- | --- | --- |
| Standard Cox regression model | 1.08 (1.05~1.11) | <0.001 |
| Two-piecewise Cox regression model | |  |
| < 8 | 1.04 (0.98,1.11) | 0.226 |
| ≥8 | 1.14 (1.10,1.18) | < 0.001 |
| *P* for the log likelihood ratio test |  | 0.027 |

Variables included in model 3 (table 2) were adjusted.

Table S3. Multivariable results by Cox regression analysis*

|  | **Model 1** | |  | **Model 2** | |  | **Model 3** | |
| --- | --- | --- | --- | --- | --- | --- | --- | --- |
|  | **HR (95% CI)** | ***P* value** |  | **HR (95% CI)** | ***P* value** |  | **HR (95% CI)** | ***P* value** |
| **28-day mortality** |  |  |  |  |  |  |  |  |
| Log2-PIV | 1.12 (1.09~1.15) | <0.001 |  | 1.11 (1.09~1.14) | <0.001 |  | 1.07 (1.03~1.10) | <0.001 |
| Quartile |  |  |  |  |  |  |  |  |
| Q1 | Reference |  |  | Reference |  |  | Reference |  |
| Q2 | 1.11 (0.94~1.30) | 0.233 |  | 1.11 (0.94~1.31) | 0.229 |  | 1.04 (0.87~1.23) | 0.687 |
| Q3 | 1.54 (1.32~1.80) | <0.001 |  | 1.54 (1.32~1.80) | <0.001 |  | 1.31 (1.1~1.55) | 0.002 |
| Q4 | 2.06 (1.78~2.39) | <0.001 |  | 2.01 (1.73~2.33) | <0.001 |  | 1.59 (1.31~1.92) | <0.001 |
| *P* for trend |  | <0.001 |  |  | <0.001 |  |  | <0.001 |
| **90-day mortality** |  |  |  |  |  |  |  |  |
| Log2-PIV | 1.12 (1.10~1.15) | <0.001 |  | 1.12 (1.10~1.14) | <0.001 |  | 1.08 (1.05~1.11) | <0.001 |
| Quartile |  |  |  |  |  |  |  |  |
| Q1 | Reference |  |  | Reference |  |  | Reference |  |
| Q2 | 1.17 (1.01~1.35) | 0.035 |  | 1.17 (1.01~1.35) | 0.282 |  | 1.10 (0.94~1.27) | 0.225 |
| Q3 | 1.63 (1.42~1.87) | <0.001 |  | 1.63 (1.42~1.87) | <0.001 |  | 1.41 (1.21~1.64) | <0.001 |
| Q4 | 2.10 (1.84~2.39) | <0.001 |  | 2.05 (1.8~2.34) | <0.001 |  | 1.68 (1.42~1.99) | <0.001 |
| *P* for trend |  | <0.001 |  |  | <0.001 |  |  | <0.001 |

Model 1 adjusted for none; Model 2 adjusted for age, sex, and race; Model 3 adjusted for age, sex, race, SOFA, SAPS II, mechanical ventilation, renal replacement treatment, vasoactive agents, myocardial infarct, congestive heart failure, hypertension, diabetes mellitus, chronic pulmonary disease, liver disease, chronic renal disease, cerebrovascular disease, temperature, respiratory rate, MAP，SPO2, WBC, HGB, BUN, anion gap, bicarbonate, Lactate， RDW， glucose，creatinine，INR.

PIV, pan-immune-inflammation value; SOFA, Sequential Organ Failure Assessment; SAPS II, Simpliﬁed Acute Physiology Score II; MAP, mean blood pressure; SPO2, peripheral capillary oxygen saturation; WBC, white blood cell; HGB, hemoglobin; RDW, red cell distribution width; BUN, blood urea nitrogen; INR, international normalized ratio.

*Patients with missing values were excluded.

Table S4 Multivariable results by Cox regression analysis*

|  | **Model 1** | |  | **Model 2** | |  | **Model 3** | |
| --- | --- | --- | --- | --- | --- | --- | --- | --- |
|  | **HR (95% CI)** | ***P* value** |  | **HR (95% CI)** | ***P* value** |  | **HR (95% CI)** | ***P* value** |
| **28-day mortality** |  |  |  |  |  |  |  |  |
| Log2-PIV | 1.12 (1.09~1.14) | <0.001 |  | 1.11 (1.09~1.13) | <0.001 |  | 1.06 (1.03~1.09) | <0.001 |
| Quartile |  |  |  |  |  |  |  |  |
| Q1 | Reference |  |  | Reference |  |  | Reference |  |
| Q2 | 1.04 (0.90~1.21) | 0.562 |  | 1.01 (0.87~1.17) | 0.893 |  | 0.98 (0.84~1.14) | 0.762 |
| Q3 | 1.40 (1.22~1.6) | <0.001 |  | 1.36 (1.18~1.56) | <0.001 |  | 1.22 (1.05~1.42) | 0.011 |
| Q4 | 1.94(1.70~2.22) | <0.001 |  | 1.86 (1.63~2.11) | <0.001 |  | 1.51 (1.28~1.79) | <0.001 |
| *P* for trend |  | <0.001 |  |  | <0.001 |  |  | <0.001 |
| **90-day mortality** |  |  |  |  |  |  |  |  |
| Log2-PIV | 1.12 (1.10~1.14) | <0.001 |  | 1.11 (1.09~1.13) | <0.001 |  | 1.08 (1.05~1.11) | <0.001 |
| Quartile |  |  |  |  |  |  |  |  |
| Q1 | Reference |  |  | Reference |  |  | Reference |  |
| Q2 | 1.11 (0.98~1.26) | 0.115 |  | 1.07 (0.94~1.21) | 0.309 |  | 1.03 (0.90~1.18) | 0.648 |
| Q3 | 1.47 (1.30~1.66) | <0.001 |  | 1.43 (1.26~1.61) | <0.001 |  | 1.30 (1.14~1.49) | <0.001 |
| Q4 | 1.97 (1.76~2.21) | <0.001 |  | 1.89 (1.69~2.12) | <0.001 |  | 1.61 (1.39~1.87) | <0.001 |
| *P* for trend |  | <0.001 |  |  | <0.001 |  |  | <0.001 |

Model 1 adjusted for none; Model 2 adjusted for age, sex, and race; Model 3 adjusted for age, sex, race, SOFA, SAPS II, mechanical ventilation, renal replacement treatment, vasoactive agents, myocardial infarct, congestive heart failure, hypertension, diabetes mellitus, chronic pulmonary disease, liver disease, chronic renal disease, cerebrovascular disease, temperature, respiratory rate, MAP，SPO2, WBC, HGB, BUN, anion gap, bicarbonate, Lactate， RDW， glucose，creatinine，INR.

PIV, pan-immune-inflammation value; SOFA, Sequential Organ Failure Assessment; SAPS II, Simpliﬁed Acute Physiology Score II; MAP, mean blood pressure; SPO2, peripheral capillary oxygen saturation; WBC, white blood cell; HGB, hemoglobin; RDW, red cell distribution width; BUN, blood urea nitrogen; INR, international normalized ratio.

*Patients with autoimmune disease, including systemic lupus erythematosus, systemic sclerosis, ulcerative colitis, rheumatoid arthritis, ankylosing spondylitis, Crohn's disease, multiple sclerosis, myasthenia gravis and polymyositis, were excluded.

Figure S1


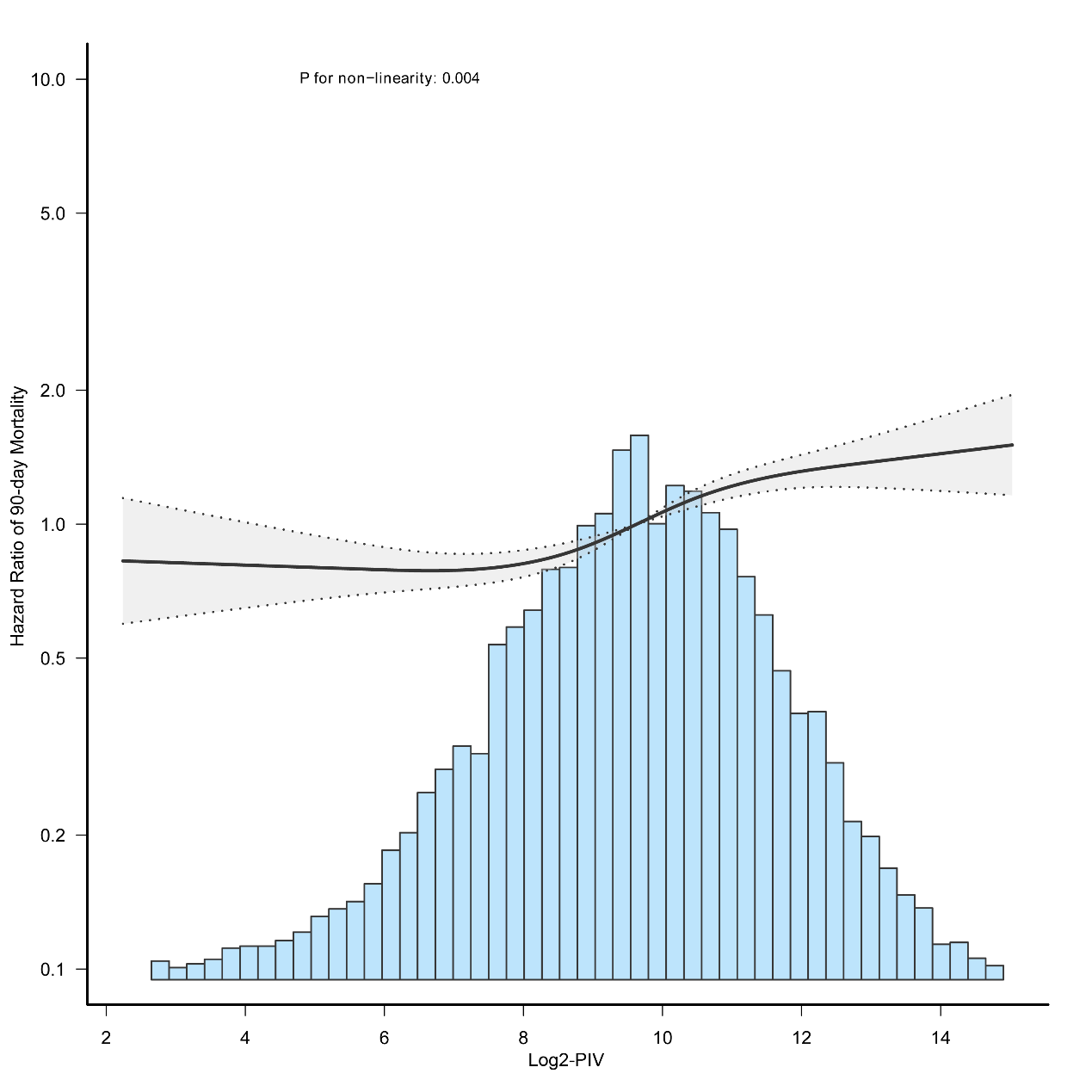


Figure S2


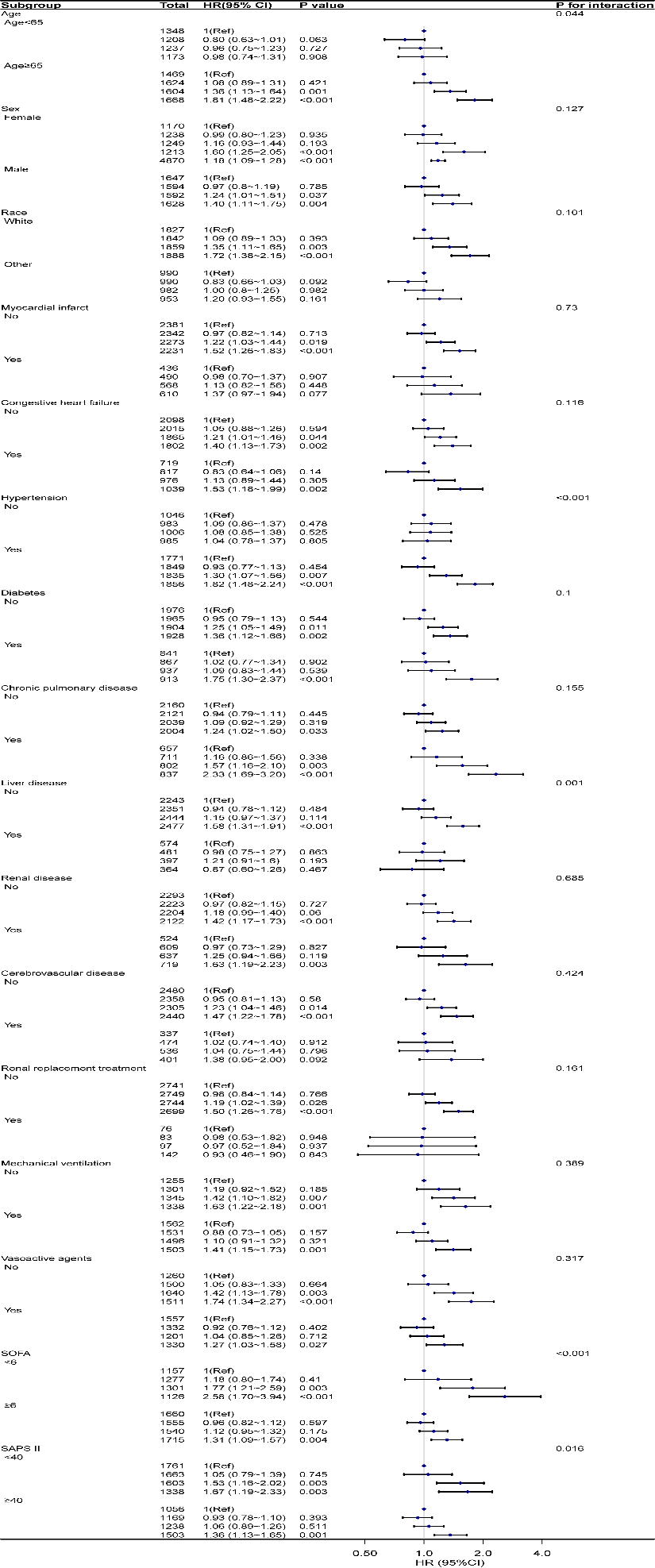


Figure S3


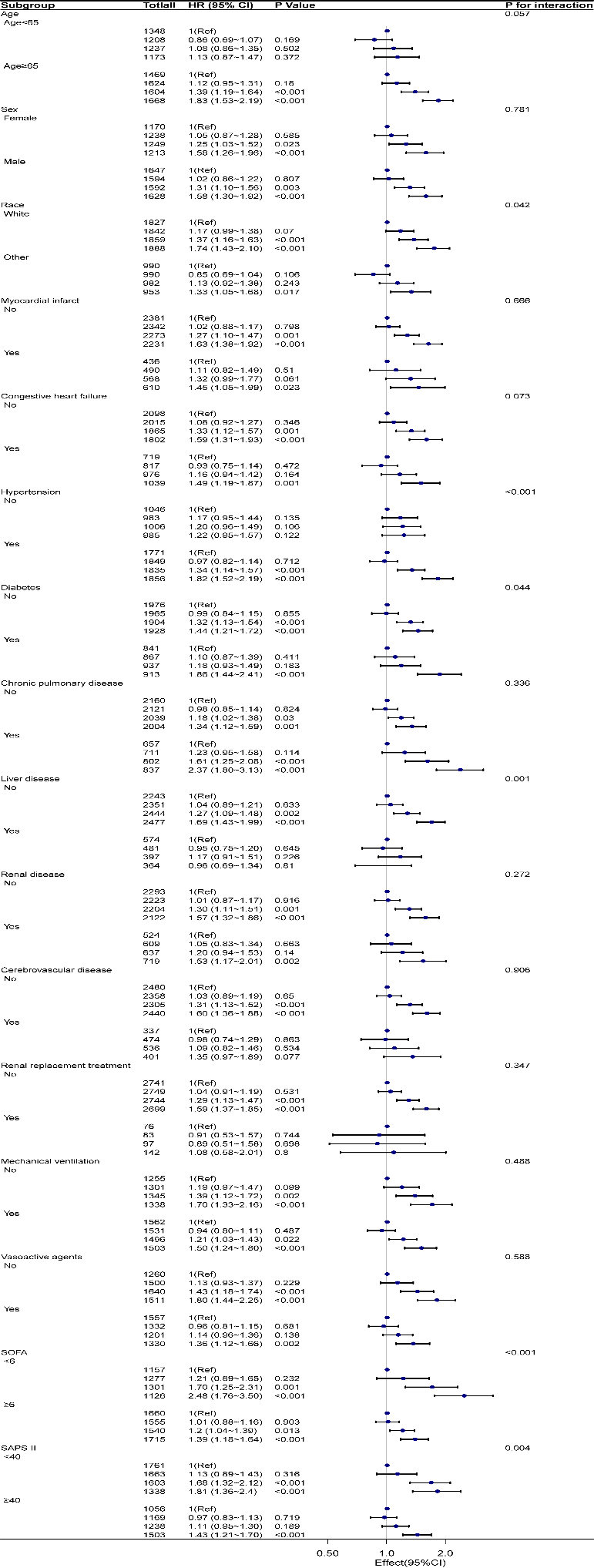

Supplement: Supplementary file 1 — Supplementary Information. [file 41598_2024_66142_MOESM1_ESM.docx]
